# 

**Published:** 1869-01-15

**Authors:** 


### *CONTENTS.*

ORIGINAL COMMUNICATIONS:

Argentine Albuminuria. Biological Society of Paris—M. Claude Bernard, Pres't........ 33

Solution of Nitro-Sulphate of Iron. By Z. C. McElroy, M D., Zanesville, O.. 41

A Case of Aconite Poisoning. By John W. Henley, M.D., Yates City, Ill... 45

Electricity: Its Use in Hysteria. By James T. Newman, M.D., Chicago..... 48

Remarks on the Prunus Virginiana, Particularly the Wine and Ferrated Wine of Wild Cherry. By X. Toner, M.D., Albany, N. Y........ 50

PROCEEDINGS OF SOCIETIES:

Wisconsin State Medical Society........ 55

EDITORIAL:

Medical Legislation—Protection........ 60

Rush Medical College........ 61

FOREIGN ITEMS:

From *La Gazette Medical*........ 62

BOOKS RECEIVED:

A Treatise on the Principles of Medicine and Pathology, Diseases of Women and Children, and Medical Surgery. By W. Paine, M.D........ 63

The Philadelphia System of Obstetrics. In Twelve Parts—Fully Illustrated. By Jos. S. Longshore, M.D., Professor of Obstetrics, etc........ 63

Clinical Lectures on Diseases of the Urinary Organs. By Sir Henry Thompson, M.D., Surgeon Extraordinary to H. M. King of the Belgians.. 63

On Chronic Bronchitis—Especially as connected with Gout, Elphysema, and Diseases of the Heart. By E. Headlam Greenhow, M.D., Fellow of the Royal College of Physicians, etc........ 63

Mackenzie on the Use of the Laryngoscope in Diseases of the Throat. Edited by J. Solis Cohen, M D........ 64

Wythe's Physicians' Pocket Dose and Symptom Book........ 64

Cleaveland's Pronouncing Medical Lexicon........ 64

RUSH MEDICAL COLLEGE—SPRING ANNOUNCEMENT........ 64

